# Open Access Target Validation Is a More Efficient Way to Accelerate Drug Discovery

**DOI:** 10.1371/journal.pbio.1002164

**Published:** 2015-06-04

**Authors:** Wen Hwa Lee

**Affiliations:** Structural Genomics Consortium, Nuffield Department of Medicine, University of Oxford, Oxford, United Kingdom

## Abstract

There is a scarcity of novel treatments to address many unmet medical needs. Industry and academia are finally coming to terms with the fact that the prevalent models and incentives for innovation in early stage drug discovery are failing to promote progress quickly enough. Here we will examine how an open model of precompetitive public–private research partnership is enabling efficient derisking and acceleration in the early stages of drug discovery, whilst also widening the range of communities participating in the process, such as patient and disease foundations.

## Abstract 2

An open access model of precompetitive public–private research partnership can reduce the risks and increase the speed of the early stages of drug discovery, while widening participation to new communities such as patient and disease foundations.

## Open Innovation: Fifty Shades of Grey?

The rate at which new drugs are being discovered has flatlined despite massive investment in research and development (R & D) and new technologies, and there is a common belief that the pharmaceutical business model might be flawed [1,2]. Despite a recent upward trend, the number of “first-in-class” therapies has not changed significantly. The fundamental problem is that our understanding of human biology and pathophysiology is too poor to be able to predict the right drug targets for the right patient populations. Therefore, the widespread aversion of institutions and public and private funders to share information prior to and after publication and the overprotection of intellectual property in order to provide return on investment are amongst the most counterproductive practices to the discovery of new medicines. This strategy is at odds with the evidence for enhancing commercial outcomes as well. Most universities lose money through their technology transfer activities [3] with some notable few exceptions. Most start-up companies do not have a patent at the outset, and it is common that current patenting activities are used to restrict and limit possible uses of underdeveloped discoveries [4]—a true Tragedy of the Anticommons.

In the last decades, the pharmaceutical industry has accessed many innovative ideas and products through mergers, takeovers, and in-licensing. This trend follows the concepts of Open Innovation, as defined by Chesbrough [5,6], in which companies improve their competitiveness by entering into open external partnerships. However, Open Innovation appears to have had very little impact on the trajectory of drug discovery, presumably because such partnerships most often only involve inward flow of knowledge or exclusive exchanges between a limited number of partners. On occasion, they involve outflow of assets, but these assets are typically restricted to a few appointed groups and with strings attached. Open Innovation as exercised above is an advance, but its true impact remains to be seen.

On the other hand, there are several initiatives in the biomedical research area that are true to the public’s understanding of the term “open” and that practice a genuine open access or open source and precompetitive scientific commons approach. These projects appear to have had significant impact, and some have been transformational, including the Single Nucleotide Polymorphisms Consortium (1999; [7]), the International HapMap Project (2002; [8]), the Open Source Malaria Project (2011; [9]), and the Structural Genomics Consortium (SGC) (2003; [10]; www.thesgc.org).

It is important to reiterate that drug discovery is a long and intricate process with different types of challenges and thus different approaches. In the early hypothesis generation stages, it makes sense for different communities to join efforts to create novel, open research tools that can be used by everyone [11]. This is in contrast to late, derisked stages, where different groups can begin development of their own proprietary products [12]. As such, we will be examining the impact of open access in early stages of drug discovery, especially in target discovery and validation, as these are strong indicators of success in creating new medicines.

## Open Access to Eliminate Choke Points in Early Target Discovery

The SGC was formed in 2003 with the open access ethos as its core tenet and has since catalysed research in new areas of human biology and drug discovery by focusing to a large extent on less well-studied areas of human biology and disease. The SGC, strongly supported by its pharmaceutical industry partners, places all its research output and reagents, including industry-standard small molecule chemical inhibitors (probes) in the public domain without restriction on use. These are used widely to interrogate protein targets and signalling pathways to further our understanding of disease mechanisms, for instance.

The establishment of a precompetitive and patent-free consortium has had many advantages; some were obvious and others unexpected. What was clear at the outset was that adhering to open access principles allowed cross-leveraging of public and private funds to explore novel areas of human biology in an organised way, thus reducing duplication and sharing the risks and costs that no single institution could bear alone. It was also clear that it would place the emphasis on the science and on accelerating the transfer of knowledge to the scientific community, rather than on commercial interests. The SGC has disseminated tens of thousands of cDNA clones and thousands of samples of several chemical inhibitors, with hardly any transactional costs. Hundreds of academic papers report the use of SGC-generated reagents, and across the pharmaceutical and biotechnology sectors, SGC reagents are used daily to advance internal drug discovery programs.

What was less appreciated was the extent to which the consortium’s position would resonate with the academic and clinical communities. The SGC collaborative network now comprises of scientists in hundreds of institutions around the world—all of whom have committed to the open access principles and who contribute their ideas and results to the public domain. The value of this collaborative network and of the network of academics making discoveries with SGC chemical probes is difficult to quantify, but a rough comparator is the fact that industry typically budgets hundreds of thousands of dollars to fund and manage even a single collaboration. Given that the SGC collaborates with over 300 different laboratories and has disseminated over 4,000 samples of chemical probes so far, one may argue that its open-access network provides hundreds of millions of dollars of value.

## Open Access Drives Faster Pioneering Science

Epigenetics is an exciting area of biology that has gained a large degree of attention over the last few years, holding a vast potential for drug discovery [13]. However, despite there being more than 400 proteins known to be involved in epigenetic regulation [14], as of 2010, only one target family, histone deacetylases (HDACs), comprised of more than 20 proteins, had been targeted by cell active inhibitors in the public domain.

Appreciating that the other proteins and protein families implicated in epigenetic regulation were likely important, in 2005, the SGC began to purify them and solve their 3-D structures. In 2009, after having made significant progress, GlaxoSmithKline (GSK) approached SGC with the “outside-the-box” idea to design highly-potent, highly-selective inhibitors of these proteins using structure-guided methods and to provide them to the community without restrictions. The concept was that these probes would be rapidly used by the community to help define the roles of the proteins in human biology, as exemplified by past experiences with similar approaches in the field of probes for nuclear receptors [11]. With funding from the Wellcome Trust and the Ontario government, and medicinal chemistry expertise from industrial partners, the project was launched and focused on underexplored protein families such as lysine demethylases (KDMs; [15–19]), histone methyltransferases (HMTs; [20–24]), and Bromodomains [25–27] (full list of SGC probes: www.thesgc.org/chemical-probes/epigenetics).

The open access model provided the framework to receive invaluable advice from scientists at GSK about the bromodomains protein family. Based on insights from GSK scientists, we initiated a collaboration on the role of the BRD4 bromodomain in NUT midline carcinoma—an incurable rare cancer. Within 11 months, a small molecule called SGCBD01 (aka JQ1) was designed, synthesised, and used to show that inhibition of BDR4 promoted both differentiation and apoptosis of patient-derived primary cells [28,29].

The rapid progress made with bromodomains is a testament to the power of open access, but the true value of open science emerged once SGCBD01 or JQ1 and additional probes by GSK (I-BET) [30] and Pfizer (PFI-1) [31] were distributed to the community. Within half a year, these compounds were used by the community to link bromodomains to septic shock [30], leukaemia [32,33], multiple myeloma [34], cardiac hypertrophy [35,36], HIV infection [34,37,38], and MYC regulation [34,38].

## Open Access Generates Pioneering Drug Programmes and Clinical Studies Quicker—for Everyone

**Fig 1:**
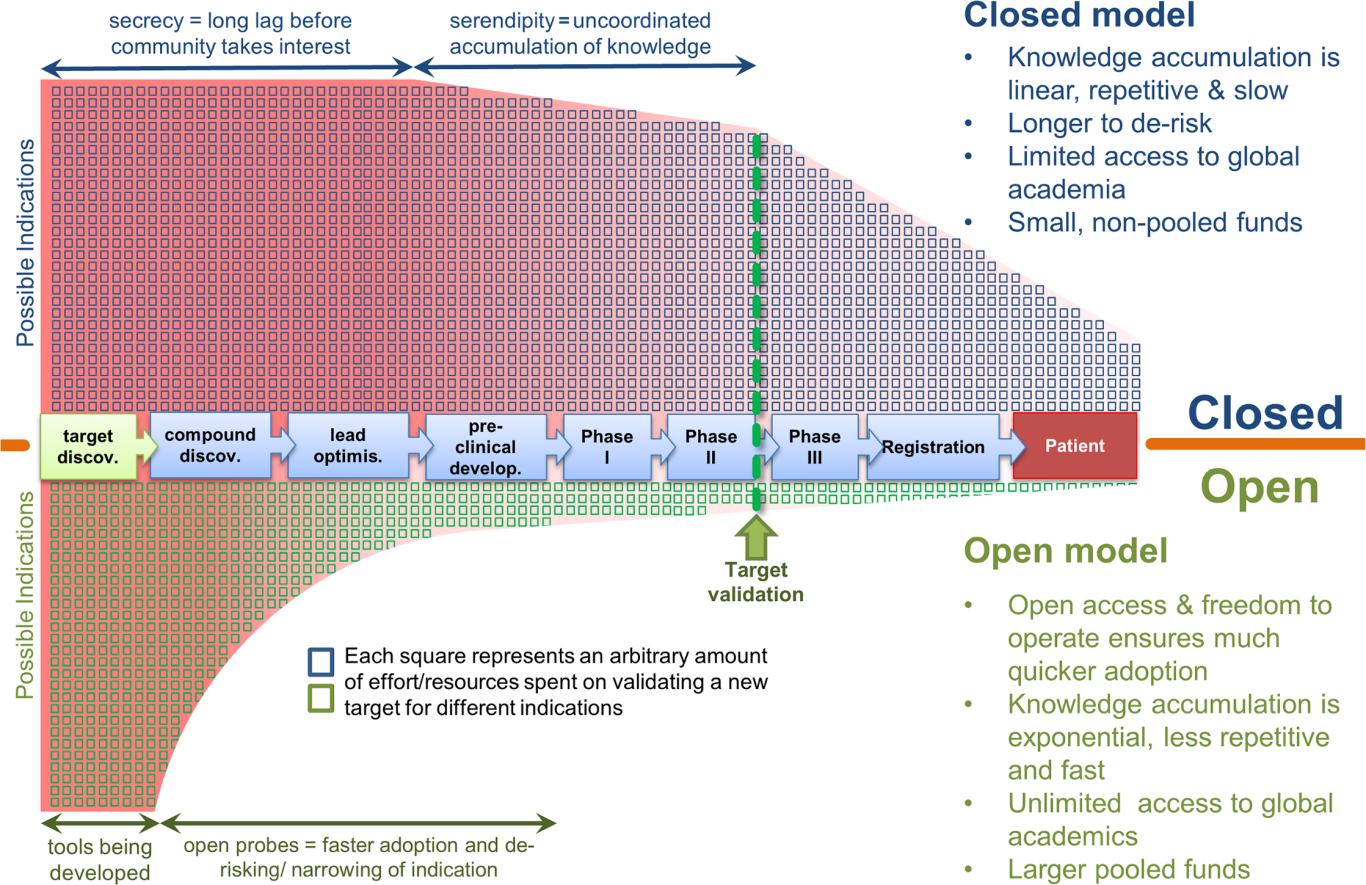
Open science accelerates identification of the best targets and drug indications, in the correct patient population. The Closed (upper half) model is compared to the Open (lower half) model; the availability of open access chemical tools for novel proteins and the freedom to operate enable the global community to explore different indications and diseases in parallel and quickly share back through publications. The breadth and depth of the studies in the open model lower the risks of failure in subsequent stages in a typical drug discovery programme, allowing the scientists to focus on the most promising indications, whilst reducing the level of effort (open squares), wastage, and duplication engendered by secrecy of the closed models.

Discoveries based on the use of small molecule probes are highly valued by industry and academia, and experience shows that availability of potent, specific, drug-like chemical compounds (probes) increases the chances of final success in drug discovery programs [11]. The probes enable the definition and validation of targets and pathways, using experimental systems that resemble final and approved therapeutic modalities, in its cellular context [39]. The aim of the SGC in producing open-access chemical probes was to spur and accelerate innovative drug discovery (Fig 1). The breadth, depth, and reproducibility of numerous studies enabled by high-quality BET bromodomains chemical probes led to the registration of the first clinical trial aimed at this class of proteins by GSK in April 2012 (Clinical Trial Registration: NCT01587703), 16 months after the seminal publication on targeting BRD4 histone reader with SGCBD01 or JQ1 (28). Currently, there are twelve clinical trials with BET bromodomains inhibitors registered (Table 1).

**Table 1:** Clinical trials targeting bromodomains and registered on ClinicalTrials.gov (National Library of Medicine and National Institutes of Health, United States), as of 1 January 2015.

NCT Number	Title	Conditions	Molecule	Sponsor or Collaborators	Phases	First Received
**NCT01587703**	A Study to Investigate the Safety, Pharmacokinetics, Pharmacodynamics, and Clinical Activity of GSK525762 in Subjects With NUT Midline Carcinoma (NMC) and Other Cancers	Carcinoma, midline	Drug: GSK525762	GlaxoSmithKline	Phase 1	3 April 2012
**NCT01713582**	A Phase I, Dose-finding Study of the Bromodomain (Brd) Inhibitor OTX015 in Haematological Malignancies	Acute leukaemia, other hematological malignacies	Drug: OTX015	OncoEthix	Phase 1	22 October 2012
**NCT01949883**	A Phase 1 Study Evaluating CPI-0610 in Patients With Progressive Lymphoma	Lymphoma	Drug: CPI-0610	Constellation Pharmaceuticals, The Leukaemia and Lymphoma Society	Phase 1	10 September 2013
**NCT01987362**	A Two Part, Multicenter, Open-label Study of TEN-010 Given Subcutaneously	Solid tumors	Drug: TEN-010	Tensha Therapeutics	Phase 1	5 November 2013
**NCT02157636**	A Phase 1 Study Evaluating CPI-0610 in Patients With Previously Treated Multiple Myeloma	Multiple myeloma	Drug: CPI-0610	Constellation Pharmaceutical, The Leukaemia and Lymphoma Society	Phase 1	28 May 2014
**NCT02158858**	A Phase 1 Study Evaluating CPI-0610 in Patients With Acute Leukaemia, Myelodysplastic Syndrome, or Myelodysplastic/Myeloproliferative Neoplasms	Acute Myeloid Leukaemia (AML), Myelodysplastic Syndrome (MDS), Myelodysplastic/ Myeloproliferative Neoplasms (MDS/MPN)	Drug: CPI-0610	Constellation Pharmaceuticals, The Leukaemia and Lymphoma Society	Phase 1	5 Jun 2014
**NCT02259114**	A Phase IB Trial With OTX015, a Small Molecule Inhibitor of the Bromodomain and Extra-Terminal (BET) Proteins, in Patients With Selected Advanced Solid Tumors	NUT midline carcinoma, triple negative breast cancer, non-small cell lung cancer with rearranged ALK gene/fusion protein or KRAS mutation, Castrate-resistant Prostate Cancer (CRPC), pancreatic ductal adenocarcinoma	Drug: OTX015	OncoEthix	Phase 1	3 October 2014
**NCT02296476**	A Trial With Dose Optimization of OTX015 in Recurrent Glioblastoma Multiforme (GBM) Patients	Glioblastoma ultiforme	Drug: OTX015	OncoEthix	Phase 1 Phase 2	3 October 2014
**NCT02303782**	A Study Assessing tOTX015 in Combination With Azacitidine (AZA) or AZA Single Agent in Patients With Newly-diagnosed Acute Myeloid Leukaemia (AML) Not Candidate for Standard Intensive Induction Therapy (SIIT)	AML	Drug: OTX015, Drug: Vidaza (azacitidine)	OncoEthix	Phase 1 Phase 2	24 November 2014
**NCT02308761**	A Dose Escalation and Cohort Expansion Study of TEN-010 in Patients With Acute Myeloid Leukemia and Myelodysplastic Syndrome	AML, MDS	Drug: TEN-010	Tensha Therapeutics	Phase 1	14 November 2014
**NCT02369029**	An Open-label, Non-randomized, Multicenter Phase I Dose Escalation Study to Characterize Safety, Tolerability, Pharmacokinetics and Maximum Tolerated Dose of BAY 1238097 in Subjects With Advanced Malignancies	Neoplasms	Drug: BAY1238097	Bayer	Phase 1	17 February 2015
**NCT02391480**	A Phase 1 Study Evaluating the Safety and Pharmacokinetics of ABBV-075 in Subjects With Advanced Cancer	Advanced cancer, breast cancer, non-small cell lung cancer, AML, multiple myeloma	Drug: ABBV-075	AbbVie	Phase 1	12 March 2015

It is interesting to note that until early 2015, nine out of ten trials (GSK’s being the exception) were registered by either a small biotech or start-up company. In one notable example, the clinical candidate (CPI-0610) used in three different trials was developed in partnership with a nonprofit, patient-driven organisation—The Leukaemia and Lymphoma Society. As such, one could argue that the open model is challenging the established system by enabling a wider range of groups to easily and quickly access innovation. This leads to a more efficient route to attract incentives and rewards, especially in the fragile interstice of “valley of death” in drug discovery.

## Open Access Invigorating Patient-Driven Research—The Most Transformative Impact

The open access model has had system-wide impact in biomedical science and drug discovery. This is tremendously exciting, but what we are now interested in exploring is whether we can make a much bigger impact if the open access model is combined with the determination and focus of patient-driven initiatives.

Open access initiatives and patient and disease foundations share a common focus—to advance science toward a cure. For most patients and their families, issues of ownership and profit are irrelevant. This holds especially true for foundations of untreatable or rare conditions such as *Fibrodysplasia Ossificans Progressiva* (FOP; “Stone Man Syndrome”) and Adult Polyglucosan Body Disease (APBD).

Could marrying the SGC open-access approach to drug discovery with the dedication and focus of disease foundations result in faster advances? We believe so.

FOP United Kingdom, the foundation supporting research into FOP, worked with the SGC and its collaborative network on structure-based development of inhibitors for ACVR1, a kinase for which specific mutation has been identified of the disease [40,41], as well as for the understanding of mutant phenotypes [42]. Molecules from this partnership are now being optimised for future clinical studies.

Most recently, the SGC and CHDI Foundation have teamed up to discover and characterise new drug targets for Huntington’s disease (HD). In this first partnership of its kind, both have explicitly agreed not to file for patents on any of the collaborative research and to make all reagents and knowledge available without restriction [43]. This is a pioneering move by CHDI and establishes a template for how patient-orientated funders can help the research community develop new drugs—in fact, the Ontario Brain Institute has already entered into a similar agreement with the SGC, to further research on Rett’s syndrome.

Finally, participation of patients and disease foundations is also pivotal for the establishment of anonymised primary cell and tissue open platforms. This will accelerate generation of robust, clinically meaningful assays to profile molecularly-targeted probes using phenotypic and biomarker readouts linking novel targets to new indications [39].

## Open Access and Potential Pitfalls

One curious behaviour is that the scientific community has been giving a disproportionately large focus on earlier tools rather than exploring other open probes available so far, probably reflecting the established risk aversion we encounter in biomedical research [44]. Therefore, even though open access can accelerate investigation of novel biology, it is important for the community to be aware of this trend and to cover more efficiently all the novel tools and target areas that are being enabled by open initiatives.

## Expanding the Open Access Ecosystem

The main tenets of precompetitive, patent-free and open-access research have enabled the establishment of a new dynamic ecosystem, not confined to biomedicine alone. The open access ethos ensures a high degree of crosstalk between sectors, including more than 300 academic groups, government agencies, biotech companies, start-ups, entrepreneurs, lawyers, economists, social scientists, and patients, all working together to expand the impact of open access drug discovery (S1 Fig). The last ten years have been exciting and rewarding with a future that looks bright and even more promising—for science, for patients, and for society as a whole; what are you waiting for? Join us!

## Supporting Information

S1 Fig The SGC super network. The SGC’s Open Access model is transformative and encourages crossfields, cross-sector interactions to accelerate drug discovery and advancement of basic biology. This has resulted in the establishment of a network of collaborations and projects, covering a wide range of initiatives implemented alongside strategic partners. For a full overview of the SGC’s scientific coverage, please refer to www.thesgc.org.
**• Human tissue platforms and Inflammation**: exploring biology of novel proteins using patient-derived primary cells and tissues.
**• Target Enabling Packages (TEPs)**: generating open access “toolkits” (structures, assays, proteins, chemical starting points, etc.) to allow exploration of novel, genetically validated targets.
**• Structural parasitology & neglected diseases**: using structure-based methodologies and science to advance development of novel treatments [http://www.thesgc.org/sddc].
**• Rare diseases**: expanding the understanding of structure and function of the associated proteins as well as the effects of disease mutations [http://www.thesgc.org/science/rare-diseases].
**• Patient & disease foundations**: working together with focused networks of disease specialists to further increase knowledge in structural biology and functional and chemical spaces around implicated proteins [Dolgin, Nat Med 2014].
**• Kinase inhibitors for human & plant sciences**: facilitating the cross-sector use of open-access chemical probes targeting basic biology [Knapp et al., Nat Chem Biol 2013].
**• Open clinical proof-of-concept**: expanding the precompetitive, patent-free model towards phase II clinical proof-of-concept [Norman et al., Sci Transl Med 2011a, 2011b].
**• Start-ups & incubators**: creation of open access toolkits for pioneer biology and dynamic entrepreneurial communities have already resulted in creation of independent start-ups.
**• Ethics & economics**: open-access model has created new paradigms around the philosophy and practical and economic aspects of discovering novel treatments and medicines.
**• Governments & policymakers:** availability of a model that can expedite drug discovery and reduce its cost is of interest for governments, always under pressure to address societal healthcare needs.
(TIF)

## Abbreviations:

AML: Acute Myeloid Leukaemia
APBD: Adult Polyglucoson Body Disease
CRPC: Castrate-resistant Prostate Cancer
FOP: Fibrodysplasia Ossificans Progressiva
GSK: GlaxoSmithKline
HD: Huntington’s disease
HDAC: histone deacetylase
HMT: histone methyltransferase
KDM: lysine demethylase
MDS: Myelodysplastic Syndrome
MPN: Myeloproliferative Neoplasms
R & D: research and development
SGC: Structural Genomics Consortium
TEP: Target Enabling Packages
