# Supplementary figures and images for: Open Access Target Validation Is a More Efficient Way to Accelerate Drug Discovery
Source: PLoS Biol. 2015 Jun 4;13(6):e1002164. doi: 10.1371/journal.pbio.1002164 (PMC4456377; doi:10.1371/journal.pbio.1002164)

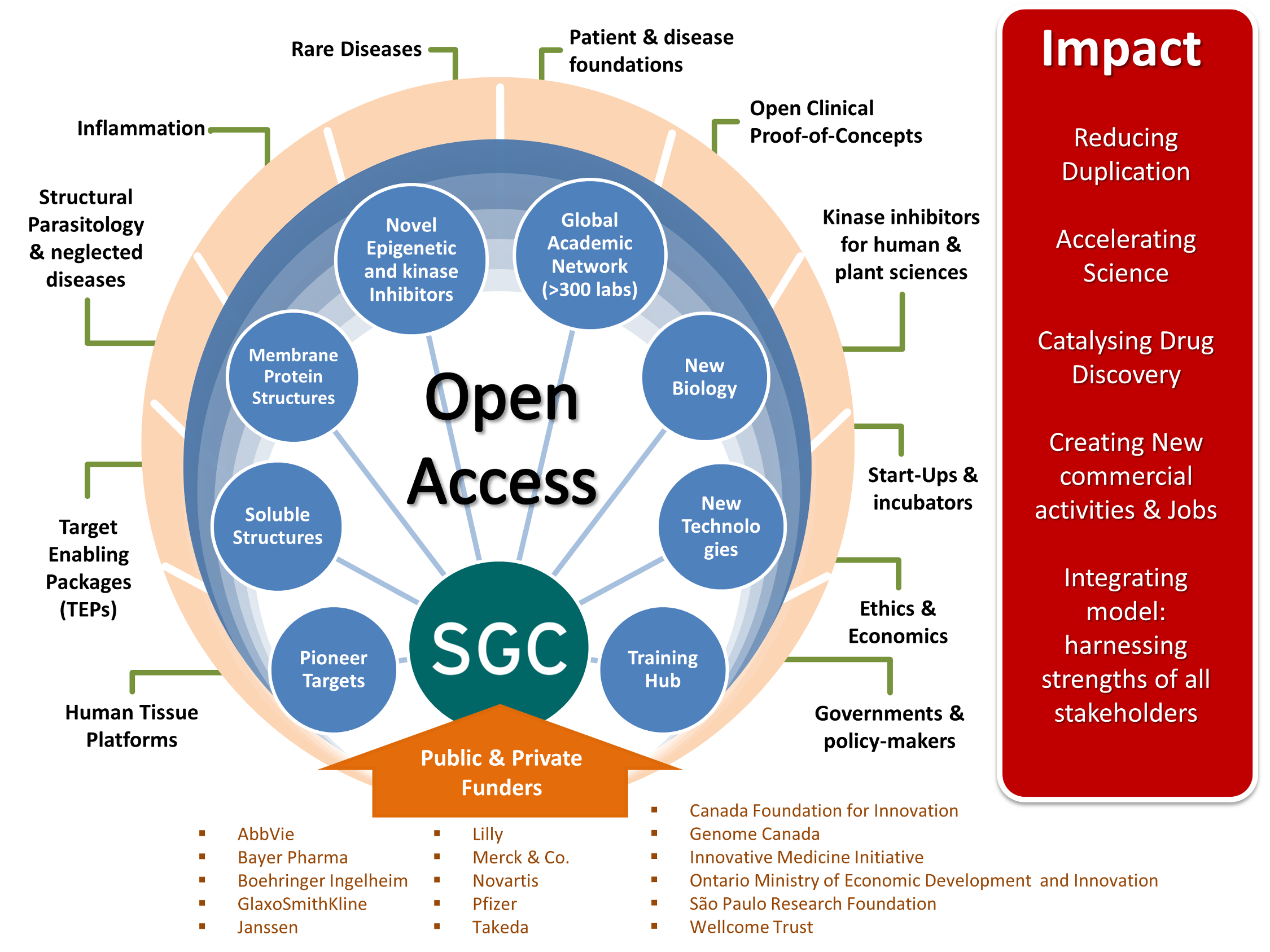

Supplement: S1 Fig — The SGC’s Open Access model is transformative and encourages crossfields, cross-sector interactions to accelerate drug discovery and advancement of basic biology. This has resulted in the establishment of a network of collaborations and projects, covering a wide range of initiatives implemented alongside strategic partners. For a full overview of the SGC’s scientific coverage, please refer to www.thesgc.org. • Human tissue platforms and Inflammation: exploring biology of novel proteins using patient-derived primary cells and tissues. • Target Enabling Packages (TEPs): generating open access “toolkits” (structures, assays, proteins, chemical starting points, etc.) to allow exploration of novel, genetically validated targets. • Structural parasitology & neglected diseases: using structure-based methodologies and science to advance development of novel treatments [http://www.thesgc.org/sddc]. • Rare diseases: expanding the understanding of structure and function of the associated proteins as well as the effects of disease mutations [http://www.thesgc.org/science/rare-diseases]. • Patient & disease foundations: working together with focused networks of disease specialists to further increase knowledge in structural biology and functional and chemical spaces around implicated proteins [Dolgin, Nat Med 2014]. • Kinase inhibitors for human & plant sciences: facilitating the cross-sector use of open-access chemical probes targeting basic biology [Knapp et al., Nat Chem Biol 2013]. • Open clinical proof-of-concept: expanding the precompetitive, patent-free model towards phase II clinical proof-of-concept [Norman et al., Sci Transl Med 2011a, 2011b]. • Start-ups & incubators: creation of open access toolkits for pioneer biology and dynamic entrepreneurial communities have already resulted in creation of independent start-ups. • Ethics & economics: open-access model has created new paradigms around the philosophy and practical and economic aspects of discovering novel treatmen [file pbio.1002164.s001.tif]
